# Paraduodenal hernias in children: Etiology, treatment, and outcomes of a rare but real cause of bowel obstruction

**DOI:** 10.1016/j.ijscr.2019.10.001

**Published:** 2019-10-10

**Authors:** Sadi A. Abukhalaf, Aya Mustafa, Mohammad N. Elqadi, Ahmad Al Hammouri, Khalil N.M. Abuzaina, Radwan Abukarsh, Ihsan Ghazzawi, Shareef Hassan, Nathan M. Novotny

**Affiliations:** a Al-Quds University, Faculty of Medicine, Jerusalem, Palestine; b Yarmouk University, Faculty of Medicine, Irbid, Jordan; c Governmental Hebron Hospital, Hebron, Palestine; d Palestine Red Crescent Society Hospital, Hebron, Palestine; e Palestine Medical Complex, Ramallah, Palestine

**Keywords:** Paraduodenal hernia, Internal hernia, Left, Pediatric, Intestinal obstruction

## Abstract

**Introduction:**

Paraduodenal Hernia (PDH) is the most common variant of internal hernia and occurs most often in males during their 4^th^–6^th^ decades of life. PDH in pediatric age group has rarely been reported in literature with only five cases of age up to 10 years were reported. PDH is a rare cause of intestinal obstruction, which may lead to subsequent strangulation and perforation of the bowel.

**Presentation of case:**

We reported a 1.5 year-old male child presented with intestinal obstruction. The patient experienced abdominal pain, vomiting and irritability. Abdominal x-ray showed distal intestinal obstruction which was discovered to be a result of left PDH incidentally during the surgery. In addition, we performed a literature search using PubMed to identify the published cases of PDH. We also compared our case with the characteristics of all reported PDHs in toddlers and children up to 10 years of age in a concise table.

**Discussion:**

Despite its congenital origin, PDH has been reported in childhood age group in very rare occasions rendering the accurate incidence of PDH in infancy and childhood unknown. PDHs can be asymptomatic or can present most commonly with recurrent upper abdominal pain. Diagnosis is quite difficult in the absence of symptoms but could be achieved using a computed tomography (CT-scan) in non-emergency symptomatic patients. Surgical repair is mandatory to avoid potential complications.

**Conclusion:**

As PDH can lead to major and life threatening complications, it must remain in our minds as a possible cause of intestinal obstruction.

## Highlights

• Paraduodenal Hernia has rarely been reported in the pediatric age group.
• Paraduodenal Hernia is a rare cause of intestinal obstruction and is often misdiagnosed.
• CT-scan is the gold standard mean for diagnosis in most cases.
• Paraduodenal Hernia must be kept in mind as a possible cause of intestinal obstruction.

## 1 Introduction

Paraduodenal hernia (PDH) is the most common type of visceral hernia although overall it is an infrequent type of hernia [1]. PDH could be congenital or acquired after a surgery or trauma. PDH was first classified into right and left PDH in 1889 by Jonnesco since each type has a different anatomic origin and embryologic development [2]. A left PDH occurs when the bowel herniates through the paraduodenal fossa of Landzert, and it is more common than right PDH [1]. PDH is an extremely rare cause of intestinal obstruction in children.

PDH accounts for 0.2–0.9% of all intestinal obstruction cases and frequently patients remain asymptomatic until a complete obstruction of the small bowel develops. PDH often presents with vague symptoms of abdominal pain, and/or vomiting [1]. PDH is challenging in diagnosis and needs a very high index of suspicion due to its rarity and non-specific presentation. Preoperative diagnosis of PDH is unusual. Abdominal CT-scan and ultrasound in context of the high clinical suspicion may lead to the diagnosis preoperatively [3]. Intestinal obstruction and strangulation of PDH can carry a high mortality and morbidity rates rendering prompt management crucial. Surgical repair is the management of choice [4]. Herein, we report a case of 1.5 year-old male presented with a small bowel obstruction, he was found to have a large left paraduodenal hernia during the operation. The work has been reported in line with the SCARE criteria [5].

## 2 Case presentation

A 1.5-year-old male patient presented to our hospital with a three day history of persistent non-bilious vomiting, associated with paroxysmal irritability, hypo-activity and decreased oral intake. His last bowel movement was three days prior to admission. The child was born full term via normal vaginal delivery following uneventful pregnancy and had no other past medical or surgical history.

**Fig. 1:**
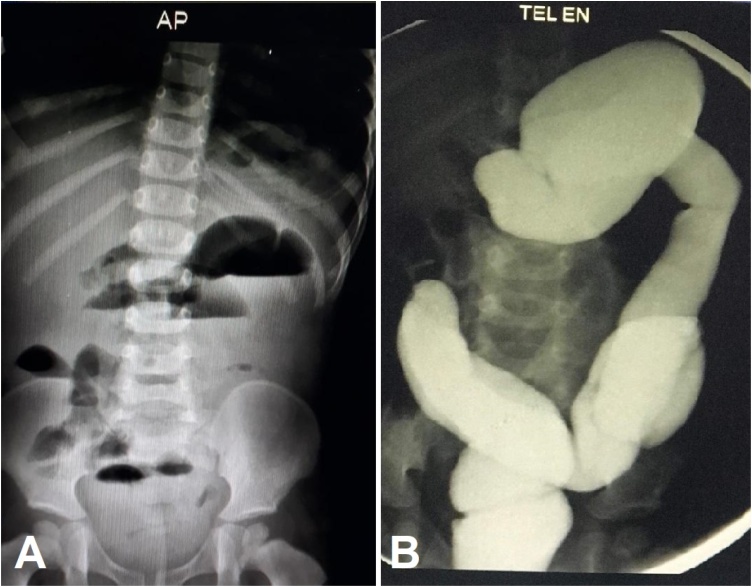
Initial radiological imaging for the child (A) Plain abdominal x-ray shows multiple air-fluid levels, and (B) Barium enema shows a complete obstruction in the mid transverse colon.

**Fig. 2:**
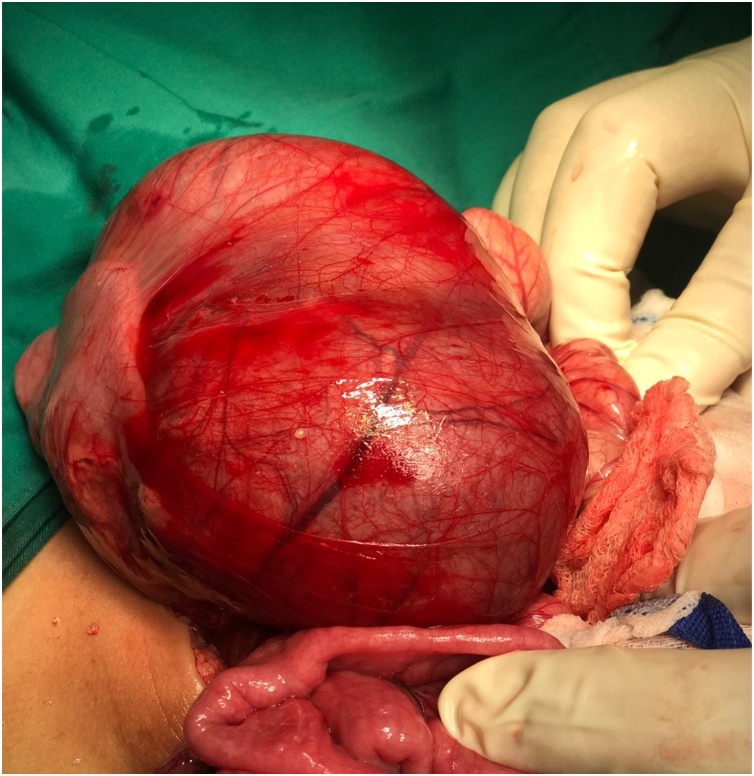
Intraoperative photograph shows the left paradudenal hernial sac.

On examination, the patient was hypoactive but alert. Abdomen was soft and non-distended with no palpable masses and intact hernaal orifices. Initial investigations revealed normal electrolytes, complete blood count, urine analysis, and urine culture. Abdominal x-ray showed air-fluid levels [Fig. 1a]. An abdominal ultrasound showed dilated fluid filled bowel loops with ileoilial intussusception which resolved spontaneously. Barium enema showed a complete obstruction in the mid transverse colon [Fig. 1b]. An urgent laparotomy revealed a large left paraduodenal hernia, with cecum, terminal ileum and right colon being incarcerated within the hernial sac without any signs of ischemia or necrosis [Fig. 2].

**Fig. 3:**
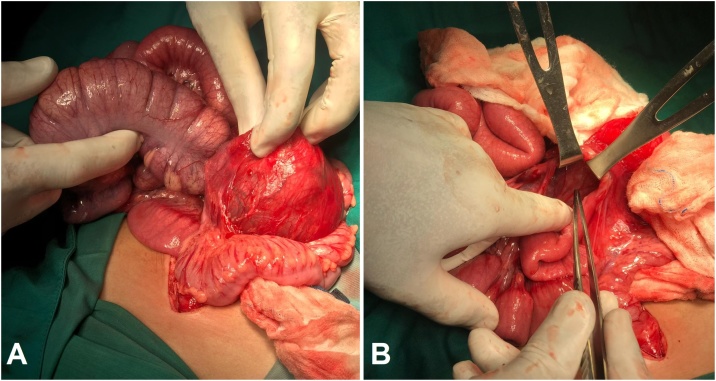
Intraoperative photographs (A) while reducing the sac contents into the abdomen cavity, and (B) closing the defect.

We managed the left paraduodenal hernia by reducing the sac contents into the abdomen cavity without bowel resection [Fig. 3a], followed by excising the sac and closing the defect [Fig. 3b]. Postoperatively, the patient did not pass stool for 3 days, a rectal examination revealed fecal impaction which was managed with normal saline irrigation. The patient started to have bowel movements on the postoperative day 5 and was discharged on the postoperative day 6.

## 3 Discussion

Paraduodenal hernia, also called paramesocolic or mesocolic hernia, is a rare anatomic anomaly and accounts for 53% of all internal hernia which is defined as a protrusion of an internal organ through an opening in intraperitoneal recess. PDH is of congenital origin and formed by the herniation of small bowel through a congenital defect. This congenital defect is a potential space behind the mesocolon, formed as a consequence of fusion failure of the mesocolon with the parietal peritoneum [6].

In general, internal hernia is three times more common in males than in females. Despite its congenital origin, PDH usually presents in patients between their fourth and sixth decades of life, in contrast to our patient who presented at the age of 1.5 years [3]. An accurate incidence of paraduodenal hernias in infancy and childhood is unknown, but quite rare [7].

Table 1 shows characteristics of the previously reported PDH in toddlers and children up to 10 years of age. Three of five patients were male with mean age of 5 years. Symptoms included abdominal pain, nausea and vomiting with chronic symptoms being experienced by three of them. Two of the patients died, they were discovered to have paraduodenal hernia during autopsy. One study has reported five cases of PDH in neonates [8].

**Table 1:** Characteristics of the previously reported PDH cases in toddlers and children up to 10 years of age.

	Case 1	Case 2	Case 3	Case 4	Case 5
Author, year	Jay Preshad, 1998 [11]	Jay Preshad, 1998 [11]	Vivian Tang, 2011 [7]	Vivian Tang, 2011 [7]	This study
Age (Years)	2.5	5	8	8	1.5
Gender	M	M	F	F	M
Presentation	Abdominal pain, mild abdominal distention and vomiting	Abdominal pain, nausea and vomiting	Chronic abdominal pain and constipation	Vomiting	Vomiting, paroxysmal irritability, hypo-activity and poor oral intake
Chronic Symptoms	None	Yes (Previous 5 visits to the pediatrician complaining of abdominal pain)	Yes (Chronic abdominal pain and constipation)	Yes (long history of vomiting)	None
Radiographic findings	Abdominal US showed SBO with small amount of peritoneal fluid	NA	UGI series demonstrated mal-rotation. With delayed radiograph showing non-specific distal SBO.	SBFT showed a cluster of loops consistent with a RPDH	Abdominal US showed dilated bowel loops. Barium enema showed a complete obstruction in the mid transverse colon
Definitive diagnosis	The autopsy showed incarcerated LPDH, and gangrenous bowel	The autopsy showed strangulated LPDH involving small bowel	Congenital RPDH, Mal-rotation	Congenital RPDH, Mal-rotation	Urgent laparotomy incidentally revealed a large LPDH
Treatment	NA	NA	NA	NA	Reducing sac contents, excising the sac and closing the defect
Complications	Bowel wall necrosis, Death	Bowel wall necrosis, Death	None	None	None

LPDH = Left Para-Duodenal Hernia, RPDH = Right Para-Duodenal Hernia, US = Ultrasound, UGI = upper gastrointestinal study, SBFT = small bowel follow-through, NA = Not Available, SBO = small bowel obstruction.

Congenital PDH is divided into three types, left PDH which accounts for 75% of the cases, right PDH accounting for the remaining 25% and the extremely rare transverse PDH [9]. The left PDH is thought to be caused by the *in utero* herniation of the small intestine through the left paraduodenal fossa of Landzert. The fossa of Landzert is located lateral to the fourth part of the duodenum, posterior to the inferior mesenteric vein (IMV) and the ascending branch of the ascending left colic artery (LCA), directly beneath the posterior parietal peritoneum. Most authors believe that LPDH result from the malrotation of the midgut, by invagination of the small bowel into the avascular segment of the left mesocolon that therefore fails to fuse with the posterior parietal peritoneum [10]. Still, some authors believe that primitive fusion failure of the left mesocolon results in a congenital fossa, where the small bowel herniates later on [9].

Between the fifth and the twelfth week of normal gestation of the human embryo, the primitive midgut undergoes a counterclockwise rotation of 270° around the axis of the superior mesenteric artery (SMA). Usually after this process, fusion of the duodenum, mesentery and mesocolon with the peritoneum of the posterior wall takes place. The Right PDH develops when the pre-arterial limb aborts its counterclockwise rotation at 180° and the post-arterial segment continues to develop and rotates normally. As a consequence the small bowel will become trapped behind and to the right of the SMA and behind the ascending mesocolon which will fail to fuse with the peritoneum of the posterior wall [9]. The extremely rare transverse PDH, also called middle mesocolic hernia, develops when the small bowel herniates through the fossa mesocolica within the transverse mesocolon [1].

Almost half of the PDH patients are asymptomatic and the other half can present with recurrent upper abdominal pain (43%) [6], symptoms of partial or complete small bowel obstruction or mesenteric vascular compromise [10]. However, 69% of the symptomatic cases present with chronic symptoms of around 1.8 years duration preceding the obstruction or strangulation symptoms. Chronic symptoms may include dyspepsia, intermittent colicky abdominal pain, and vomiting [9].

Nonspecific findings can be found during physical examination, including abdominal distension and tenderness [3]. In addition, left paraduodenal hernia can lead to the appearance of a palpable abdominal mass in the left upper quadrant only in one third of cases [6].

Many of the PDH are diagnosed intraoperatively, at autopsy or during radiological investigation for unrevealed complain. Therefore, the pre-operative diagnosis of asymptomatic PDH resembles a diagnostic challenge. Whenever symptoms develop, diagnosis made easier for clinician [6]. Plain x ray is the first line radiological exam in the emergency department which may reveal the PDH as a cluster of small bowel loop in the right or left upper quadrants with signs of small bowel obstruction [4]. In the absence of abdominal emergency, Computed tomography remains the gold standard imaging modality for early diagnosis of this clinical entity which typically appears as a mass of small bowel between the stomach and the pancreas [11]. Other imaging modalities that can be used include ultrasonography and barium-enhanced studies such as upper gastrointestinal series [4].

Once PDH is diagnosed, surgical repair is mandatory to avoid the increased risk of incarceration or strangulation which accounts for the 20–50% mortality rate [6]. Surgical repair involves reduction of the hernia contents manually (enlarging the defect, if it is needed to reduce the contents), resection of the sac and closing the defect with continuous or interrupted suture [6]. While closing the defect, care must be taken not to injure the adjacent mesenteric vessels, particularly the inferior mesenteric vein [4].

## 4 Conclusion

PDH is challenging in diagnosis due to its rarity and non-specific presentation. It causes intestinal obstruction leading to serious consequences which can be fatal. Therefore, a high index of suspicion and early intervention are essential.

## Sources of funding

No funding or grant support.

## Ethical approval

The study is exempt from ethnical approval in our institution.

## Consent

The patient consent was obtained by the infant’s father. And the father accepted the final edition of the article.

## Author’s contribution

Study concept or design: Radwan Abukarsh, Ihsan Ghazzawi, Shareef Hassan.

Data collection and data analysis: Sadi A. Abukhalaf, Aya Mustafa, Mohammad N. Elqadi.

Writing the paper: Sadi A. Abukhalaf, Ahmad Al Hammouri, Khalil N.M Abuzaina, Nathan M. Novotny.

## Registration of research studies

This is a case report study. No need for registration.

## Guarantor

Dr. Sadi A. Abukhalaf.

## Provenance and peer review

Not commissioned, externally peer-reviewed.

## Declaration of Competing Interest

The following authors have no financial disclosures: Sadi A. Abukhalaf, Aya Mustafa, Mohammad N. Elqadi, Ahmad Al Hammouri, Khalil N.M Abuzaina, Radwan Abukarsh, Ihsan Ghazzawi, Shareef Hassan, Nathan M. Novotny.
